# In their own words: Perspectives of IPV survivors on obtaining support within the healthcare system

**DOI:** 10.1371/journal.pone.0310043

**Published:** 2024-09-06

**Authors:** Anita S. Hargrave, Carol Dawson-Rose, Dean Schillinger, Fiona Ng, Jessica Valdez, Amanda Rodriguez, Yvette P. Cuca, E. Hayes Bakken, Leigh Kimberg

**Affiliations:** 1 Department of Internal Medicine, University of California San Francisco (UCSF), San Francisco, California, United States of America; 2 Division of General Internal Medicine, San Francisco General Hospital, University of California San Francisco (UCSF), San Francisco, California, United States of America; 3 Department of Community Health Systems, School of Nursing, University of California San Francisco (UCSF), San Francisco, California, United States of America; 4 Division of General Internal Medicine, Center for Vulnerable Populations, University of California San Francisco (UCSF), San Francisco, California, United States of America; 5 UCSF Institute for Health Policy Studies, University of California San Francisco (UCSF), San Francisco, California, United States of America; 6 Department of Obstetrics, Gynecology & Reproductive Sciences, University of California San Francisco (UCSF), San Francisco, California, United States of America; 7 Division of General Pediatrics, Children’s Hospital of Philadelphia, Philadelphia, Pennsylvania, United States of America; Public Library of Science, UNITED KINGDOM OF GREAT BRITAIN AND NORTHERN IRELAND

## Abstract

**Background:**

Almost half of all women in the US experience intimate partner violence (IPV) in their lifetime. The US Preventive Services Task Force recommends IPV screening paired with intervention for women of reproductive age. We aim to understand clinical practices and policies that are beneficial, detrimental, or insufficient to support survivors of IPV in a safety-net healthcare system.

**Methods:**

We sampled 45 women who were 18–64 years old, had experienced IPV within the prior year and were patients in the San Francisco Health Network. We conducted in-depth, semi-structured interviews to elicit their perspectives on disclosing IPV and obtaining support within the healthcare system. We analyzed our data using thematic analysis and grounded theory practices informed by ecological systems theory.

**Findings:**

We identified four themes regarding factors that impeded or facilitated discussing and addressing IPV across interpersonal and systemic levels relating to relationship-building, respect, autonomy and resources. (1) Interpersonal barriers included insufficient attention to relationship-building, lack of respect or concern for survivor circumstances, and feeling pressured to disclose IPV or to comply with clinicians’ recommended interventions. (2) Interpersonal facilitators consisted of patient-centered IPV inquiry, attentive listening, strength-based counseling and transparency regarding confidentiality. (3) Systemic barriers such as visit time limitations, clinician turn-over and feared loss of autonomy from involvement of governmental systems leading to separation from children or harm to partners, negatively affected interpersonal dynamics. (4) Systemic facilitators involved provision of resources through IPV universal education, on-site access to IPV services, and community partnerships.

**Conclusions:**

Women experiencing IPV in our study reported that relationship-building, respect, autonomy, and IPV-related resources were essential components to providing support, promoting safety, and enabling healing in the healthcare setting. Successful trauma-informed transformation of healthcare systems must optimize interpersonal and systemic factors that improve survivor wellbeing while eliminating barriers.

## Introduction

Intimate partner violence (IPV) is defined as physical, sexual, psychological or economic violence and/or stalking by a current or former intimate partner [1,2]. It is associated with long-term negative effects on health and social functioning, including increased chronic pain, depression, post-traumatic stress disorder (PTSD), gastrointestinal or genitourinary disorders and decreased social connectedness [3–5]. IPV also occurs in the context of structural violence which further limits resources and safety options [6]. Structural violence is generally thought of as the ways in which people are harmed by societal structures [7–9]. For instance, many people experiencing IPV (‘survivors of IPV’) have limited income [6,10,11]. Societal factors such as the high cost of housing, hiring discrimination, and insufficient economic policies to support IPV survivors can restrict their financial independence and force them to stay with an abusive partner [6,10,11]. Healthcare staff have the opportunity to provide needed support and resources to people experiencing IPV, in addition to treating the physical or psychological sequalae of violence [5,12]. In 2018, the United States Preventive Services Task Force (USPSTF) recommended routine screening for IPV among reproductive-age women [13]. However, it remains unclear whether survivors are benefiting from this practice [14]. Further, implementing healthcare screening and intervention programs requires systems-change and a trauma-informed approach [15–17]. To better care for survivors, we need a deeper understanding of their experiences disclosing IPV and receiving support.

In the United States (US), almost half of all women have experienced intimate partner violence and the prevalence of IPV is highest amongst marginalized and minoritized communities [1]. The safety-net healthcare system in the US provides healthcare to low-income populations, including people who are uninsured, underinsured, and/or use Medicaid. The safety-net system covers a large portion of the American population, as 32% of working-age adults are uninsured or underinsured [18–20]. The safety-net disproportionately serves people from marginalized communities [18–20] and provides inpatient, emergency and outpatient ambulatory services [19,20]. Patients in the safety-net healthcare system suffer from high burdens of IPV and structural violence, which synergistically cause harm [15,21–23]. As such, it is important that safety-net healthcare staff and clinicians adopt interpersonal practices and advocate for systemic changes that promote safety, facilitate healing and are informed by best practices for supporting IPV survivors with marginalized identities [24–27].

Although the USPSTF has recognized that IPV is a significant public health concern, the best way to care for patients with a history of IPV in primary care remains under debate [13,27,28]. It has been proposed that screening for IPV may be one means of preventing and mitigating the impact of IPV if it is followed by appropriate resources [25,29]. As a result, there have been increasing calls from the US government, professional medical organizations and public law (i.e., the Affordable Care Act) to implement IPV screening into clinical settings, particularly for women of childbearing age [13,15,30–32]. However, there is also growing evidence that IPV disclosure rates are below the actual prevalence of IPV [33,34] and concern that screening followed by referral is not a sufficiently robust model for addressing IPV in primary care settings [14,35].

Due to the high prevalence of IPV and structural violence among women who receive their healthcare in the safety-net system, we sought to understand what current clinical practices and structures within the healthcare system are perceived as beneficial, detrimental, or insufficient to support IPV survivors. Using qualitative methodologies, we explored the narratives of women experiencing IPV who received care in the San Francisco Health Network (SFHN), the largest safety-net healthcare network in San Francisco. We analyzed the interpersonal dynamics between patients and clinicians as well as the systemic factors that might influence the healthcare experiences of survivors. This research has the potential to inform best practices in providing supportive and effective care to patients experiencing IPV.

## Methods

The Aspire to Realize Improved Safety and Equity (ARISE) evaluation was a longitudinal, mixed-methods cohort study designed to evaluate the impact of a multi-faceted IPV quality improvement (QI) initiative on women experiencing IPV in order to inform further healthcare innovations [25]. Participants were patients in at least one of seventeen SFHN clinics (5 hospital campus clinics and 12 community clinics) who identified as (cis-gender or transgender) women aged 18 to 64. Participants were included if they reported having experienced IPV within the 12 months prior to enrollment as described during the informed consent process; however, they did not need to have disclosed their experiences of IPV to a healthcare clinician. Participants were excluded if they were not fluent in English, Spanish, or Cantonese or could not provide informed consent. We obtained verbal consent using a ‘teach-to-goal’ process [36]. Research assistants documented the participants’ verbal consent in a password secured database. The study was approved by the institutional review board of the University of California, San Francisco.

We recruited participants through three methods including, 1) clinician referral of patients experiencing IPV to the ARISE study using a template in the electronic health record, 2) ARISE study staff recruitment of patients after sharing a brief IPV education module on an iPad prior to clinic visits, and 3) through flyers in clinics and on the main hospital campus for direct-to-patient recruitment. Most participants were recruited through self-referral after seeing a flyer.

Between October 2017 and March 2018, we enrolled 56 participants to the ARISE study cohort using convenience sampling. All participants completed the baseline questionnaire and 45 participants completed the qualitative interviews, which represent the data for our current analysis. Three bilingual research assistants trained in qualitative methods conducted interviews over the phone and in-person. Interviews lasted approximately 60–90 minutes. We sent participants a $40 gift card after the interview. We used semi-structured interview guides, which focused on experiences of violence, survival and safety strategies, modes of coping and healing, and interactions with the healthcare system. We created the interview guide based on extant literature and expert opinion. However, given the semi-structured nature of the interviews, the trained research assistants could create new questions or adjust the interview questions depending on the participants’ responses. We audiotaped, professionally transcribed and translated interviews verbatim. We stopped collecting data once 45 interviews were completed and determined that we had reached thematic saturation through our analytic process. We de-identified the transcripts of patient information and digitally stored them in a password protected program.

We used an iterative approach for our codebook development which was primarily inductive but also utilized deductive reasoning to draw from previous literature and expert opinion regarding important codes to include [37]. Consistent with grounded theory, our inductive approach used open coding to identify new or unexpected concepts from the data by thorough reading of the transcribed interviews [38]. We incorporated these grounded theory approaches to generate findings that were grounded in the data and that derived meaning from the behaviors and perspectives of the participants [38]. Study investigators (LK, CDR, DS, EH, BA) read five transcripts to discuss and identify preliminary codes. All study investigators reviewed and developed consensus about the codes to create the codebook. Next, three coders independently double-coded five interviews (AH, FN, JV) and met regularly with the Co-PI (LK) to apply the codebook and establish inter-coder reliability. Coding discrepancies were reconciled via consensus. Throughout the coding process, the coding team (AH, FN, JV, LK, CDR) held regular meetings to continue the iterative process and ensure we maintained inter-coder reliability. We used Dedoose Qualitative Data Analysis Software to code the interviews.

We used thematic analysis to analyze and organize our data into overarching impressions and important findings related to IPV and healthcare using previous described methods [37]. Our thematic analysis was further informed by ecological systems theory, which posits that the environment may influence an individual’s behavior to a varying degree over time [39]. We used the framework of ecological systems theory to understand our data in the context of interpersonal and systemic factors related to interactions within the healthcare system. We created detailed summaries after coding each interview using a “theoretical memo,” which captured initial thematic impressions of the coders [40]. To ensure research triangulation, we held meetings with the analytic team to reach a consensus regarding key themes, data presentation and analysis. The analytic team consisted of clinicians, medical trainees, and experts in IPV, communication and qualitative methodologies. In our analytic meetings, we reflected on our positionality and the ways in which our identities and life experiences influenced our interpretation of the data [38].

## Results

A total of 45 participants completed our interviews. On average they were 43 years old. Approximately a third (36%) identified as Black or African American, 24% as White and 13% as Multiracial. A little over one-quarter (27.3%) reported that they were Hispanic or Latinx. All participants identified as cis-gender women and most reported their partner was male (98%). Further participant characteristics are shown in Table 1.

**Table 1:** Sociodemographic information N = 45.

Sociodemographic characteristics and life experiences N (%)	
**Age yrs. (mean, SD)** ^a^	43.0 (SD 12.4)
**Race** ^b^	
**•** American Indian/Alaska Native	1 (2.2%)
**•** Asian	1 (2.2%)
**•** Black or African American	16 (35.6%)
**•** More Than One Race	6 (13.3%)
**•** White	11 (24.4)
**Ethnicity**	
**•** Hispanic/Latinx	12 (27.3%)
**•** Not Hispanic/Latinx	32 (72.7%)
**Participant Gender Identity** ^c^	
**•** Female	45 (100%)
**•** Transgender Female	0 (0%)
**Partner Gender Identity**	
**•** Female	1 (2.2%)
**•** Male	44 (97.8%)
**Education**	
**•** 11^th^ Grade or Less	12 (26.7%)
**•** High School or GED	8 (17.8%)
**•** 1–3 Years of College/Associate Degree	20 (44.4%)
**•** College Graduate	5 (11.1%)
**Preferred language**	
**•** English	42 (93.3%)
**•** Spanish	3 (6.7%)
**Recruitment Method**	
**•** Clinic/Clinician Referral	1 (2.3%)
**•** iPad	6 (13.6%)
**•** Flyer	31 (70.5%)
**•** Other ^d^	7 (13.6%)

^a^ Two participants did not report their age.

^b^ We have included “American Indian and Alaska Native” as a race category in concordance with the San Francisco Health Network classifications; however, acknowledge that it is more accurately a description of tribal/political identity. There were 10 participants with “unknown/not reported” information about race: 8 of them identified as “Hispanic/Latinx,” 1 as “Not Hispanic/Latinx” and 1 did not report their ethnicity.

^c^ Participants were asked for their gender identity with the available options: Female and transgender female.

^d^ “Other” included 6 participants who were recruited through “word of mouth” or were recommended to the study by prior participants and 1 participant had missing data.

We identified four overarching themes regarding IPV survivors’ experiences in the healthcare system: (1) IPV survivors perceived **interpersonal barriers** to healing care when clinicians failed to invest in relationship-building, expressed skepticism or lack of concern regarding their experiences of IPV, and did not demonstrate respect for survivor knowledge and autonomy; (2) Conversely, **interpersonal facilitators** to obtaining support included patient-centered inquiry about IPV, attentive listening, genuine concern, strength-based counseling and discussing confidentiality; (3) Interpersonal factors were negatively impacted by **systemic barriers** such as visit constraints, clinician turn-over, lack of racially or culturally concordant clinicians, and healthcare system connection to the criminal legal system; (4) **Systemic facilitators** that were beneficial to survivors included interventions that increased privacy and Universal Education about IPV resources in clinic, facilitated connection to rapid services, and integrated community partnership. IPV survivors experienced interpersonal and systemic factors as intertwined, both when describing barriers to helpful and healing care as well as facilitators. For our results, we discuss interpersonal barriers (Section I) and facilitators (Section II), followed by systemic barriers (Section III) and facilitators (Section IV) to survivors obtaining support in the healthcare system. In each section, we organize our data into sub-themes pertaining to relationship-building, respect, autonomy and resources.

### I. Interpersonal barriers relating to relationship-building, respect, and autonomy

#### Relationship-building

Investing in relationship-building and fostering rapport with patients were important aspects of providing quality care for survivors. A participant explained that not taking the time to build rapport with a patient could be an important barrier to disclosure:

*(P32): “You just don’t go up to someone and straight up ask [about IPV]. [A patient’s] definitely not going to just talk to anyone that’s just pushy or pressuring you to talk about it. For myself, if I’m not in there to get seen for my mental health, it’s like please, just try to target whatever the issue is at the moment. You can do that [asking about IPV] at the end, but you might want to make a connection with somebody and ask them how they feel, how they’re doing, first”*

*(26 years old*, *Hispanic/Latina woman)*.

Participants believed that inquiring about IPV without first taking the time to make a personal connection with the patient and address their medical concerns could be detrimental. Survivors also expressed that patients did not want to feel pushed to disclose more information about the IPV than they were ready to divulge.

#### Respect

Participants expressed that respect for the IPV survivor’s experience and expertise was essential to garnering support in the healthcare system. Many women identified that if a clinician was dismissive or dubious of their IPV disclosure, they were much less likely to experience the healthcare setting as safe or healing. These participants feared being assumed *“to be hysterical”* and explained that *“even though [survivors’] stories sound unlikely*, *some very strange things are taking place” (P08; 55-year-old*, *non-Hispanic White woman)*. Women also voiced that clinicians often failed to understand the complexities of their intimate relationships and how their racialized and cultural identity affected this complexity. One African American participant explained:

*(P26) “I’m not getting down really like hard with the last therapist. It’s like, ‘oh, go on with your life.’ But you have to realize being an African American woman is so totally different…because we were the backbones of our men, and now we’re like nothing”*

*(59-year-old non-Hispanic Black/African American woman)*.

Although this survivor did not describe the racialized and/or ethnic background of the therapist, she implied that her experience and expertise as an African American woman—and the sociopolitical factors that affected her experiences, decision-making and options—were not understood by clinicians. Participants explained that the sociopolitical context, disrespect, and disregard for other people who have survived IPV influenced their interpersonal conversations about IPV with their clinicians. One woman referenced Christine Blasey Ford’s accusations against a Supreme Court nominee:

*(P49): “I probably much don’t talk about [IPV] to anybody and pretend that everything’s just fine because I don’t think anybody else can handle it… If people are so concerned about false allegations, then are they very surprised that a lot of survivors of sexual assault and other kinds of abuse don’t ever come forward?… In cases of sexual assault–‘he’s innocent until proven guilty’ translates to ‘she’s a liar until proven truthful.’ Why would anyone come forward when Dr. Ford came forward. Never mind the other two people who came forward, but Dr. Ford did. She not only had death threats afterward, but the President mocked her in front of the whole county to applause”*

*(33-year-old*, *Multiracial woman)*.

This participant worried that if high-profile disclosures of abuse, made by a survivor who holds many privileged identities, were called false allegations and resulted in negative consequences for the survivor, then other survivors would be treated similarly if they were to disclose their experiences of IPV.

#### Autonomy

Similarly, many patients did not feel that clinicians were prioritizing their preferences or autonomy. Survivors voiced not wanting to participate in interventions that they did not see the benefit of, or thought would be detrimental to their wellbeing. Women reported that they did not want to be pressured into treatments for common sequela of IPV such as medications for depression, post-traumatic stress disorder (PTSD) and anxiety, psychotherapy, and methods of birth control or family planning.

Participants described that some healthcare clinicians did not demonstrate appreciation for the complexity of their situation or violated their autonomy and agency around important healthcare decisions. Some participants who became pregnant while experiencing IPV conveyed that they felt coerced into moving forward with a decision regarding their pregnancy options, which was deeply harmful to them. A participant from California reported that she was 13 weeks pregnant when she was told that she was too late to terminate a pregnancy; abortion is legal in California until the physician considers the fetus viable, which is usually 24–26 weeks after becoming pregnant [41–43]:

*(P32): “When I went to the doctor’s, that’s when I found out that I was three months’ pregnant, and at the time I was, like, oh, God, I can just terminate [the] pregnancy. And then my doctor told me, ‘You’re already past the time it’s safe to do that. You can’t do that anymore.’ So, I just accepted it. And, you know, it just gave me more stress because he physically hurt me during the time I was pregnant”*

*(26-year-old*, *Hispanic/Latina woman)*.

In contrast, another participant voiced feeling pressured to move forward with terminating a desired pregnancy:

*(P02): “I even cried, and I’m like, ‘I really don’t want this.’ And she was like, ‘I mean, you’re already here now, and it’s a quick procedure.’ Like, there’s no turning around. Like, ’cause honestly, even with the [IPV] situation I was in, I really did want my child”*

*(25-year-old*, *non-Hispanic Black/African American woman)*.

In both participant experiences, the clinician did not consider the relationship between the participants’ pregnancy and the violence they were experiencing.

### II. Interpersonal facilitators relating to relationship-building, respect, and autonomy

#### Relationship-building

Participants described multiple ways that some healthcare clinicians prioritized and were skilled at relationship-building, such as demonstrating deep respect and regard for their patients’ expertise, complex challenges, and strengths. Women identified several clinician characteristics and behaviors that made them feel comfortable talking about IPV. Participants frequently reported that working with clinicians who were kind, knowledgeable, professional, and nonjudgmental helped them to share and process experiences of IPV in clinic visits. A participant explained: *“My provider was really awesome*. *So*, *I decided to be pretty candid and just share the outline of the situation of abuse” (P49; 33-year-old*, *non-Hispanic Multiracial woman)*. Specific clinician behaviors that participants associated with these characteristics included verbal and non-verbal expressions of genuine concern for patients’ wellbeing and deeply attentive listening. These gestures and actions helped to build a strong, uplifting, and empowering patient-clinician relationship—and could positively influence discussions of health and IPV.

*(P09): “[Talking about IPV] made me feel like I almost had power. And I knew she was listening because of the expressions on her face…Her words meant so much to me, to hear somebody say, ‘I’ll make time for you.’ You really are concerned about me. And that’s what started building my relationship with Dr. X… I love Dr. X, and that is the reason why I came back for medical health”*

*(unreported age*, *non-Hispanic*, *Black/African American woman)*.

Several women voiced an appreciation for strength-based counseling that focused on their strengths and validation that the IPV was not their fault. These women found it helpful when clinicians expressed that they would support the women longitudinally over time and used words of empowerment to counteract any perceived loss of power and/or control resulting from the relationship with their partner. A woman explained the importance of focusing on positivity and accomplishments particularly at the end of the visit, which helped her to leave the clinic in a better mindset.

*(P25): “[My clinician] always kind of threw in there a positive thing that I was accomplishing or that she noticed, to help me realize that I was doing good…She would never let it be negative and then have me leave the room feeling bad or anything about what I was talking about. She always made me feel really good before I left”*

*(31-year-old*, *non-Hispanic White woman)*.

#### Respect

Although the participants did not want to be forced to talk about IPV, many believed that clinicians should inquire about it in a way that respected and centered patient preferences. They highlighted that patients may not think to talk to their clinicians about abusive relationships unless they are asked about them. Women thought this was particularly true of people experiencing psychological abuse, who may be under the impression that clinicians only treat the physical harms of abuse. Participants expressed that talking about IPV with their healthcare clinician could be important because it would connect survivors to trauma-related resources and counseling. Women also believed that IPV had a significant impact on health and therefore it was essential for clinicians to know about it.

*(P12): “Yes, I’ve discussed things like [IPV] with my healthcare provider because it affects my health a lot. I have pancreatitis and it tends to upset my pancreas when I’m going through it with him. When I opened up about what was going on, the conversation was helpful. Just to have somebody to listen to me and to bounce some ideas off of—and it was somebody that I trusted, my primary caregiver. So, it was a weight lifted off my shoulders when I told her”*

*(46-year-old*, *non-Hispanic Black/African American woman)*.

Some women expressed anticipatory concern around revealing important life details to therapists and other healthcare clinicians, particularly healthcare professionals who were White. However, once participants witnessed evidence that they would be treated respectfully, some they felt that the barrier of having to work with a White clinician rather than work with a racially, ethnically, and culturally concordant individual, was not insurmountable.

*(P34): “I was like, ‘I’m not doing therapy. I don’t need no therapy. I’m not talking to no White people. I’m not telling my business…’ But she was very open. She had a lot of wisdom, and she just helped me see the things I couldn’t see for myself. They diagnosed me with PTSD, and I never knew what that really was until they explained it to me”*

*(60-year-old*, *non-Hispanic Multiracial woman)*.

#### Autonomy

Demonstrating respect for a survivor’s autonomy and agency involved being transparent about limits of confidentiality. Women often felt more comfortable talking to clinicians about or getting care related to IPV if the clinicians proactively discussed patient confidentiality and privacy. Many patients feared that their information would be shared with other patients and/or their partner. One participant explained that she did not want to get therapy for IPV because she worried that her private information would be shared with another patient who she knew was seeing the same therapist. Once the referring primary care clinician explained patient confidentiality protections, the patient was willing to go to the therapy.

*(P09): “That’s why I didn’t want to talk [to the therapist]–I didn’t want to have her to tell X, because she knows her, and they talk. And then that’s when Dr. X [the referring doctor] was like: ‘no, that can get our license pulled faster than anything…This is patient/doctor confidentiality…that’s what we’re built on. If it was something like a child was in danger, or my life was being threatened, they are mandated reporters for certain situations. If you’re uncomfortable with that then we’ll find someone else.’ I was like okay, well, if that’s the case, then I will talk with [the therapist] and see what she has to offer”*

*(unreported age*, *non-Hispanic*, *Black/African American woman)*.

Women reported feeling empowered and in control of their medical information after their clinicians explained confidentiality and privacy protections for patients.

### III. Systemic barriers relating to relationship-building, autonomy and resources

#### Relationship-building

Systemic factors are the underlying policies, practices or beliefs that influence the way that a healthcare organization functions. Therefore, by definition, they are pervasive and have a myriad of effects, including on interpersonal dynamics [44]. When participants discussed systemic barriers to safety and healing, they described how these barriers simultaneously affected relationship-building, respect, and autonomy. Participants highlighted that the very structure of the healthcare system was detrimental to relationship-building. Given the amount of time allotted for the clinical visit, many participants did not believe their clinicians had sufficient time to build strong relationships, discuss IPV, or provide necessary resources; therefore, the survivors would not discuss IPV in their visits. Several participants were frustrated with long wait times to get an appointment with a clinician and in the waiting room prior to seeing the clinician. As one participant explained: *“They have me wait a whole hour just to be seen*. *By the time I talked to the doctor*, *I’m already upset*. *That’s totally disrespectful*. *I have my children with me*. *After that*, *I said*, *‘I don’t want to go there’” (P02; 25-year-old*, *Non-Hispanic Black/African American woman)*. Many participants reported that frequent clinician turn-over resulted in destruction of the therapeutic bond and frequent re-disclosure their trauma to a new, unfamiliar clinician.

*(P46): “After I threw out all my thoughts, my feelings and all that, [the clinician] left me. And then I got a lady that was there, and she listens. Just when I get a connection and feel comfortable, she can’t do it anymore. So, I’m like, that’s not good…They don’t want to have a mental health program there. And they need it”*

*(55 -year-old*, *non-Hispanic Black/African American woman)*.

Participants reported needing access to continuous and robust mental health support.

#### Autonomy

Women voiced that a significant barrier to obtaining support from the healthcare system was fear that disclosure would lead to involvement of Child Protective Services (CPS) and/or the criminal legal system. The collaboration between the healthcare system and these regulatory systems could lead to unwanted harm to the intimate partner and/or separation from their children. Survivors of color particularly voiced distrust of the healthcare system and lack of healthcare clinicians who were racially or culturally concordant. One participant explained that she did not talk about IPV to White clinicians because she had been taught by her mother that it would result in separation from her children:

*(P33): “You don’t talk about what goes on at home. It doesn’t matter if you’re getting beat up. You don’t go telling White people your business because usually they come and remove the kids most of the time”*

*(33-year-old*, *Hispanic/Latina woman)*.

This participant identified as a Latina woman and shared that she had experienced childhood sexual molestation, severe IPV, loss of custody of her son to her abusive partner, and additional trauma. Despite these experiences, she did not seek therapy until she was 30 years old, due to the “*mentality of*, *‘Shh*, *don’t talk’”* she was taught throughout her childhood.

Although some women appreciated reassurance that their disclosure of IPV would not be shared with other patients and/or their partner, many worried about limitations to this confidentiality due to state mandatory reporting laws that could trigger unwanted engagement with law enforcement, incarceration or harm to the participant or their partner. A survivor expressed regret that her partner had been incarcerated due to IPV and therefore she would not talk to her primary care doctor about abuse:

*(P43): “That’s why I didn’t talk to my doctor or my primary about it [IPV]. Because they do report things to the police. Because I never wanted him to go to jail. But I don’t want him to hit me”*

*(44-year-old*, *non-Hispanic Black/African American woman)*.

Participants knew that healthcare and criminal legal systems were closely connected through legal reporting laws; however, they wanted their autonomy and agency to be respected within these systems. They desired transparent disclosure of when or how CPS and the police would be contacted by clinicians and direct involvement in the reporting process if it were to occur. Women wanted clinicians to respect their knowledge, the complexity of their situation and their parent-child bonds. A survivor explained that when she disclosed IPV to a clinician at a mental health clinic, he told her that he would place a report to CPS with her present. However, he called CPS without her which broke her trust in this clinic, and she did not return for ongoing care.

*(P49): “Out of a desire to be extra precautionary and careful, [the clinician] wanted to call up CPS. Said we’d make the call together, and then went ahead and did it without me. I was terrified. I’m like ‘You’re potentially sacrificing my son’s stability with me for that?’ I’m being as safe with him as I can, and you said we’d talk about it first.’ Luckily, the case got closed down but it completely destroyed any trust I had in X clinic and so I stopped working with them”*

*(33-year-old*, *non-Hispanic Multiracial woman)*.

Further, participants noted that IPV survivors are held accountable to make major life changes and comply with healthcare or CPS recommendations, but that the person perpetrating the violence is not. A woman explained that when CPS completed their investigation they would hold her responsible for the safety of the child rather than her partner, who was the source of the violence. She recounted that she would take her son on trips when her partner’s behavior started to become violent to make sure her son was not harmed.

*(P49): “[My partner] was the one who was doing the bad behavior; [CPS] didn’t care about him. They were investigating me because I was the safe parent. So, they wanted to know was I keeping my son out of [my partner’s] harm…[CPS] would give me the responsibility”*

*(33-year-old*, *non-Hispanic Multiracial woman)*.*S*

#### Resources

Structural factors also limited access to resources. Some women requested access to onsite, rapid, longitudinal resources provided within the healthcare system. Yet, in most cases, these resources were not readily available. A participant recounted that she had gone to a clinic counselor about IPV and was told that the clinic had limited resources to support her.

*(P08): “She was a counselor and a therapist for the clinic. She even said, ‘there’s not a lot of options that you have.’ I told her about all the people I tried to reach out to, and she even acknowledged, [saying] ‘I know it’s not very helpful in your situation’”*

*(55-year-old non-Hispanic White woman)*.

Participants identified the need for additional services embedded within the clinic that would assist with childcare, housing, case management, confidential counseling, and peer support groups.

### IV. Systemic facilitators relating to relationship-building, respect, and resources

#### Relationship-building

Relationships between the healthcare system and community-based Domestic Violence (DV) organizations not only helped some women to obtain immediate, trauma-informed support but also mitigated mistrust that survivors had in the medical system. A participant explained that when an advocate from a community IPV organization came to the clinic to see her after she had disclosed her IPV experience to her clinician, she felt encouraged that the clinic was taking her abuse seriously.

*(P49): “I found it really encouraging that the hospital took [IPV] so seriously that they had somebody there [from the community IPV organization] to be a support. I was hopeful that it meant that they would be able to help other women who had far fewer resources than me. I also felt that it was nice to be acknowledged that way and validated… I felt like I could trust [the clinicians] slightly more because [the community IPV organization] had never made me feel like they were going to CPS with my stuff; they made me feel like they were going to help me themselves”*

*(33-year-old*, *non-Hispanic Multiracial woman)*.

For this participant, partnerships between community organizations and the medical system could help meet survivor needs, bolster trust and improve the healthcare experience.

#### Respect and consideration

Many women noticed and valued a welcoming clinical environment that respected the distinct needs and wishes of IPV survivors. Particularly, they highlighted the importance of having kind and accommodating clinical staff in addition to their primary clinician or therapist. They felt it was respectful and de-stigmatizing to provide information on IPV to all patients regardless of disclosure. Women appreciated when clinics were considerate of the potential for contact with their partner during clinical visits and therefore created systems to minimize the risk, such as sitting in a private room instead of the waiting area before seeing the clinician or changing to a telephone visit.

*(P43): “Yeah, sometimes I be nervous because I think I’m going to see [my partner] when I leave my house. But I just go straight on through [without waiting]… [The clinician] also said, we can make arrangements to talk over the phone if I didn’t want to come in”*

*(44-year-old non-Hispanic Black/African American woman)*.

#### Resources

Women advocated for having informational material on IPV resources available and visible throughout the clinic, for all to see regardless of whether they disclosed IPV. A few women experienced seeing an immediately available IPV advocate from a community-based domestic violence agency co-located on the hospital campus. One woman recalled how important it was for her to be connected to a community IPV advocate when she disclosed her experiences of abuse to her clinician because it made her feel well cared for.

*(P49): “Then, there was my doctor who connected me with that [IPV] advocate. It felt like the doctors were all listening, and they all had a bunch of resources at their disposal, and they all made use of them and tried to get us whatever we needed to help”*

*(33-year-old*, *non-Hispanic Multiracial woman)*.

Having access to a range of general and individualized resources that were embedded in the clinic and rapidly available to survivors facilitated safety and recovery from IPV.

## Discussion

In this qualitative study of women experiencing IPV, we examined their varied experiences obtaining support and healing in the healthcare system. Our results feature the perspectives of women who were receiving healthcare from a safety-net healthcare system where there is high prevalence of IPV and patients face the synergistically adverse impacts of IPV, historical and current marginalization, and structural violence [1,7,9,15,19,20]. We identified interpersonal- and systemic-level factors that enhanced or detracted from supporting survivors in the healthcare system that contained themes relating to relationship-building, respect, autonomy, and resources.

Consistent with previous studies, we found that although many participants believed it was appropriate to ask about IPV in the healthcare setting, there were important interpersonal dynamics that influenced these conversations [45,46]. Participants explained that they wanted respectful patient-centered inquiry into IPV that included establishing rapport and eliciting patient priorities for the visit prior to being asked about IPV. Many of the study participants did not disclose IPV to their healthcare clinicians, in line with research across the globe that demonstrates that IPV disclosure rates to formal services are likely lower than the true prevalence [33,34,47–49]. In recognition of the complex reasons for low disclosure rates and the reality that most IPV survivors disclose IPV only to family and friends, IPV experts have advocated for an approach called “Universal Education” in which all patients are educated about the impacts of IPV on safety and health, and provided IPV resource information regardless of disclosure [5]. It is postulated that this approach may also result in IPV prevention or earlier intervention [5,35]. Further, many participants did not want clinicians to pressure them into pursuing interventions. IPV often includes dimensions of conflict and control [50] in which physical, psychological, economic or family planning tactics are used to control an intimate partner [5]. Several of our participants reported similar experiences of conflict and loss of control with their clinicians regarding their disclosure of IPV, medical treatments, and reproductive health. It is essential for clinicians to eliminate these parallel dynamics of power and control in clinical practice.

Survivors of IPV reported gaining more support from the healthcare system if they worked with clinicians whom they described as non-judgmental, kind, and empathic. Several participants identified specific body language (e.g., eye contact, facial expressions), undivided attention, genuine concern, and making time to deeply listen to the patient as important clinician actions that made them feel well cared for. Favorable clinician communication has been associated with increased patient engagement in IPV interventions [51]. Given that certain primary care-based IPV interventions that incorporate elements of empowerment can benefit patients [5,52] further education on patient-preferred communication strategies may improve care for survivors.

Women frequently highlighted that clinicians’ behaviors could facilitate healing by focusing on accomplishments and strengths while discussing IPV with a patient, suggesting that clinicians caring for survivors should utilize strength-based counseling. Strength-based counseling was developed as a psychological approach to understand behavior and guide people through challenging experiences by examining their strengths and the individual, community and societal factors that give life meaning [53,54]. Participants highlighted that strength-based counseling strategies may be particularly useful at the end of the visit, to help the patients transition to a more positive mindset before leaving the clinic.

Although these interpersonal dynamics were important to patients, they were also influenced by system-level factors that could limit or undermine the potential positive impact of relationships. Time constraints in clinical visits have been identified as a barrier to discussing IPV with a healthcare clinician [55]. Our participants often reported that they had insufficient time in the appointment to discuss IPV. Clinician turn-over also led to loss of a therapeutic bond and required participants to re-disclose their trauma to multiple clinicians. Every year >11,300 primary care clinicians will leave their current practice, many due to burnout related to systems-level factors (i.e., large patient panels, lack of patient access to services, documentation/administrative work) [56,57]. As such, improving care for IPV survivors requires changing local, state, and federal policies in order to increase the size of the primary care clinician workforce, prevent clinician burnout, reduce clinician turn-over and improve transitions to new clinicians [58]. IPV survivors are often isolated by their partners’ controlling behaviors. Closely co-locating and co-training multidisciplinary team members, such as social workers and mental health professionals, on how to provide high quality care for IPV survivors can reduce isolation and expand the IPV survivor’s circle of support [15,35,59]. These changes require health systems level quality improvement initiatives [15].

Survivors referred to distrust of the healthcare system due to lack of available healthcare clinicians who were racially or culturally concordant. Racism is endemic in healthcare and affects the composition of the healthcare workforce and clinical practice [60–65]. Some study participants mentioned anticipating disrespect and mistreatment by “White people,” which was a deterrent to care-seeking and disclosure of intimate life details. Minoritized and marginalized people have largely been excluded from healthcare careers [66], yet higher proportions of Black clinicians, language concordant care and workforce diversity have been shown to improve population health outcomes [67–69]. Providing IPV survivor-centered care requires major changes and diversification in the healthcare workforce [59,65]. Programs that incorporate Community Health Workers (CHWs) who are from the same communities as patients or have lived experience of IPV are an underutilized and promising approach to IPV prevention and intervention [70–72].

As seen in previous literature, some participants identified that the connection between the healthcare system and the criminal legal system, including Child Protective Services (CPS), was a significant systemic barrier to accessing healthcare, disclosing IPV and building relationships with healthcare clinicians [14,27,73–75]. These participants requested increased transparency regarding patient confidentiality and mandatory reporting, as well as active involvement in the process of reporting, if it were to occur. In the US, most states (all but four) require that healthcare professionals report IPV-related injuries. There are additional state-dependent mandatory reporting laws for child and dependent adult (“elder”) abuse or neglect, crime-related injuries and firearm-related injuries [75]. In the state of California, healthcare practitioners are mandated to submit a report to law enforcement if they are providing medical services for a physical wound or injury that is known to or suspected to be the result of assaultive or abusive conduct or a firearm. They are also required to report any concurrent child or elder abuse [76,77]. Women expressed that they preferred enhanced safety and preservation of family bonds, rather than criminalization of their partners’ behavior or involvement with CPS. These participants’ reflections are concordant with growing evidence supporting restorative or transformative justice policies and approaches to addressing IPV [78,79]. There has been a long history of leadership from Black women and IPV survivors, raising concerns about and objections to the criminalization of IPV [74,80–83]. Specifically, they have warned that criminalization contributes to mass incarceration without increasing safety [74,84–87]. Our survivors’ concerns regarding the connection of healthcare to the criminal legal system aligns with evidence showing that fear of law enforcement involvement negatively affects access to care [88–90]; mandatory reporting may not be effective in preventing violence and may be associated with harms [75,91]; and criminalization and incarceration may not prevent future violence [86,92–94].

Some participants feared or experienced loss of custody of their children. This was a deterrent to relationship-building with and disclosure to their healthcare clinicians. Additionally, loss of custody was described as a process through which “White people” remove children of color from their families. In California, where our participants reside, approximately half of all Black (46.8%) and Native American children (50.2%) have been investigated for allegations of neglect; these rates of CPS involvement are more than twice those of White children [95]. The disparate rates of referral to CPS are tied to the history and current manifestations of racism that lead to targeting of Black, Native American, and other caregivers of color for referral to CPS and can result in poverty and lack of basic resources necessary for health and wellness of children and families [96–98]. There is a growing movement to prioritize investing in resources that support children and families to survive and thrive, rather than involving CPS, and building systems of support that prevent separation of children from their parents [97–99].

In contrast, some of the participants were enthusiastic about systems-level innovations that involved community partnerships and rapid IPV Advocacy. Co-located IPV advocates can be important connections for those experiencing IPV and link them to local resources. Participants described how these services enhanced their relationships with the healthcare team, validated respect for the complexity of their situations and were powerful sources of support. Identification of IPV through screening followed by off-site referral or brief intervention may be insufficient for many women [5]. Rather, strong partnerships between healthcare systems and community organizations or integrated systems with co-location of IPV advocates, behavioral health clinicians, social workers, substance use treatment and medical-legal services to provide immediate support are likely more advantageous [15,25,59,100,101]. Women in our study confirmed that they would like to have readily available information on IPV resources in public and private spaces, which would indicate that clinicians were prepared to talk about IPV.

### Limitations

Although our work has important insights into improving the healthcare experience of IPV survivors, we acknowledge several limitations. Our participants all identified as cis-gender women. However, IPV also impacts people who identify as sexual and gender minorities and this is a woefully understudied area [102–104]. Although our recruitment did not exclude these populations, we recruited participants from existing clinical settings and did not do targeted outreach to the LGBTQ+ community. Studies have shown that people with transgender and nonbinary identities face many barriers to obtaining care and therefore may not have been in the areas of recruitment [105]. This research also did not focus on cis-gender men because IPV is more prevalent among women and more often involves severe negative health outcomes for women [5]. However, IPV also negatively impacts many men and is an important ongoing area of research [14]. Most participants recruited by tablet-based education or clinician referral received counseling about IPV prior to the study, which may have influenced their interview responses. However, participants (70%) were primarily recruited by flyer. While our study was conducted in one safety-net healthcare system, it included seventeen safety net clinics within this system. Given how prevalent IPV is, we believe that our findings can apply to many primary care clinics across the country.

### Clinical implications

**Fig 1:**
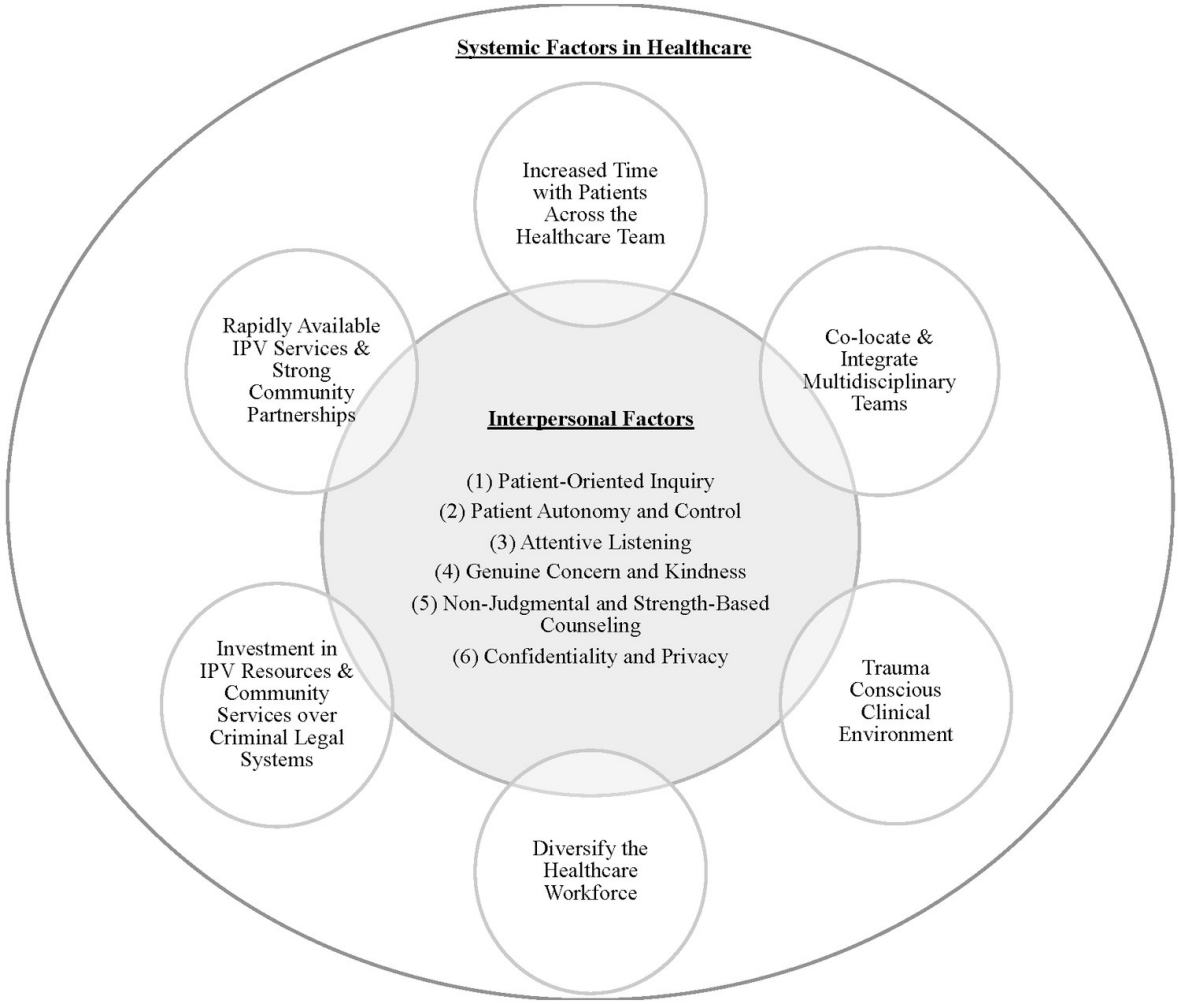
Interpersonal and systemic interventions and recommendations.

Our findings have several clinical implications regarding interpersonal and systemic factors in healthcare that influence obtaining safety and healing for survivors of IPV. Although we present our recommendations in separate tables, these factors interact with one another to shape the patient experience (Fig 1). We outline six ways in which clinicians can optimize the interpersonal experiences of discussing, addressing, and processing IPV with their patients. These interventions include (1) using patient-centered inquiry; (2) ensuring patient autonomy and control; (3) employing attentive listening; (4) expressing genuine concern and kindness; (5) using non-judgmental and strength-based counseling and (6) explaining patient confidentiality, privacy, and mandatory reporting (Table 2).

**Table 2:** Recommended interpersonal interventions.

(1) Patient-oriented inquiry
• Build rapport prior to asking about IPV
• Elicit the patient’s concerns and address the patient’s priorities as well as the IPV
• Do not pressure the patient to disclose IPV or additional details about IPV
• Provide Universal Education to all patients to help prevent and mitigate the impact of IPV regardless of disclosure
(2) Patient autonomy and control
• Foster autonomy and agency by offering options as well as respecting and supporting patient decisions
• Acknowledge relationship and life complexities
• Do not push patients to follow your recommendations—including avoid advising patients to leave a relationship
• Advocate for patients who are experiencing structural violence and center anti-racist praxis
(3) Attentive listening
• Practice deep listening while the patient is sharing their experiences of IPV
• Use body language and non-verbal cues to show the patient that you are listening (e.g., facial expressions indicating concern, eye contact with the patient rather than looking at the computer screen)
• Partner with health interpretation and other healthcare staff to foster an attentive, survivor-centered clinical environment
(4) Genuine concern and kindness
• Bring your authentic voice to expressions of concern for the patients’ wellbeing and inquire about and discuss how IPV is affecting their overall health
• Reassure patients that you believe their experiences of IPV after a disclosure and thank them for sharing
• Validate that the IPV experiences are not the patients’ fault and that no one deserves to be treated badly
• Convey that you see the patient as a whole person rather than their medical conditions or their IPV experiences
(5) Non-judgmental and strength-based counseling
• Counsel patients on clinic and community resources
• Use non-judgmental language while counseling patients
• Reflect on the patient’s strengths, passions, and sources of inspiration, hope or happiness during the visit
• End the visit with a positive reflection to leave the patients in a better mindset
(6) Confidentiality and privacy
• Before asking about or discussing IPV, explain patient confidentiality under the Health Insurance Portability and Accountability Act (HIPAA) and limitations to the confidentiality such as mandatory reporting laws in your local area
• Explain to patients what you will document in their medical record, who will have access to the information and why they will have access to it
• If mandatory reporting is required, explain the process of contacting and involving governmental or legal agencies and what information will be shared with them
• When reporting to governmental agencies, include the patient in the process and advocate for the patient’s autonomy and agency and the importance of the survivor parent/caregiver-child bond

We also recommend six ways to intervene in the healthcare system to improve the care of IPV survivors and facilitate successful integration of the interpersonal factors described above: (1) Invest in the primary care system in order to increase time with primary care clinicians, CHWs and others on the healthcare team, minimize wait times in clinic and optimize longitudinal relationships between patients and the healthcare team; (2) Co-locate and integrate multidisciplinary clinicians into clinical care including a CHW workforce, especially of people with lived experience surviving IPV; (3) Create a clinical environment tailored to the needs of survivors by ensuring all staff are equipped to form respectful relationships with and support the autonomy of IPV survivors; (4) Diversify the physician and other healthcare staff workforce, specifically increasing inclusion of people from communities under-represented in medicine; (5) Advocate for enhanced investment in IPV resources and community services rather than involvement of the criminal legal system or CPS; (6) Provide onsite, rapidly available IPV services and form strong partnerships with community IPV and violence prevention programs (Table 3).

**Table 3:** Recommended systemic interventions.

(1) Healthcare team time with patients
• Allow for longer visits with the healthcare team to discuss IPV with patients
• Reduce patient wait times prior to the visit and optimize time in clinic for the patient to speak with counselors, social workers, community health workers or DV advocates
• Create patient panel sizes that accommodate for patient trauma, complexities and care needs
• Prioritize system level approaches that address and prevent clinician burnout and minimize turn-over
• Create systems that make the transition to a new clinician easier for patients
(2) Co-locate and integrate multidisciplinary teams
• Develop an integrated system with co-location of medical-legal services, housing resources and social services within or near the clinic
• Build a community health workforce that includes people with lived experience surviving IPV
(3) Trauma conscious clinical environment
• Educate all staff about the prevalence of IPV and the importance of making the clinical environment a safe place for survivors
• Provide Universal Education on IPV prevalence, dynamics and resources to all patients
• Place informational material on resources for IPV in public and private areas of the clinic
• Create mechanisms to support the needs of survivors such as increasing privacy, minimizing potential exposure to their partner and offering spaces of solace
• Create and compensate a patient advisory committee that includes survivors, to give feedback on how the clinic can best support their needs
(4) Diversify the healthcare workforce
• Hire a diverse healthcare team to provide the option of racial, ethnic, and language concordant care
• Increase inclusion of people from communities under-represented in medicine on the healthcare team
(5) Invest in IPV resources rather than criminal legal system
• Advocate for enhanced investment in resources and community services to address IPV
• Use a patient-centered approach to involving the criminal legal system including increased transparency with patients about mandatory reporting
• Optimize referral to IPV advocacy and support services rather than involving the criminal legal system or CPS, if possible and preferred by the patient
(6) Rapidly available IPV services and strong community partnerships
• Procure an IPV advocate or staff member with expertise in IPV counseling who is embedded within the clinic for rapid referral at time of disclosure
• Per patient preference, allow community partners to see and/or speak with the patient at time of disclosure
• Support community partners and compensate them for their time
• Develop strong and longitudinal relationships with community IPV or violence prevention programs
• Learn from and with community partners regarding how to • support patients experiencing IPV

## Conclusions

Intimate partner violence persists as a major public health concern in the US, and IPV survivors continue to make difficult decisions about where to obtain safety and support while navigating complex social situations and clinical concerns. IPV survivors in our study described how the healthcare system can be an important resource for IPV survivors and can help address or prevent the health sequalae of IPV. However, they also described intersecting interpersonal and systemic factors within the healthcare system that present significant challenges for survivors and impede their ability to benefit from associated interventions. Implementing trauma-informed care may improve healthcare experiences and outcomes for survivors of IPV [16]. The key principles of trauma-informed care are grounded in understanding the extensive impact of trauma, recognizing signs of trauma, promoting healing, and creating environments that are supportive and not traumatizing for patients or clinicians [16,21,54]. Our findings underscore that fostering trauma-informed care transformations in the healthcare system must include dismantling systems or practices of care within it that are detrimental to survivors while strengthening the aspects that they find beneficial. Based on our research, we give recommendations on interpersonal and systemic interventions that could improve healthcare experiences for IPV survivors. However, we recognize that there may be barriers to implementing them in clinical practice. Future research evaluating the implementation of these recommendations in primary care settings is needed to advance survivor-centered practices and policies.

## Supporting information

S1 File Interview guide. Semi-structured interview guide for the ARISE Study.
(PDF)
