# Supplementary material for: In their own words: Perspectives of IPV survivors on obtaining support within the healthcare system
Source: PLoS One. 2024 Sep 6;19(9):e0310043. doi: 10.1371/journal.pone.0310043 (PMC11379214; doi:10.1371/journal.pone.0310043)
Supplement: S1 File — Semi-structured interview guide for the ARISE Study. (PDF) [file pone.0310043.s001.pdf]

Qualitative

Study ID ______________________

1. Last time we talked I asked you some questions about how you see yourself. I heard you say you:

a. I generally accomplish what I set out to do.
b. I have a positive attitude about myself
c. When I make plans, I am almost certain to make them work
d. I am usually confident about the decisions I make
e. I am often able to overcome barriers
f. I feel I am a person of worth, at least on an equal basis with others
g. I see myself as a capable person
h. I am able to do things as well as most other people
i. I feel I have a number of good qualities

Tell me about a time you did this.

2. Sometimes patients talk to others about their relationship. I want to hear about a time you told someone about your relationship other than me. Tell me about a time you told someone about your relationship.

Additional prompts:

The first time you went to them, what was going through your mind? What were you thinking?
What about your family? What about your friend groups? Something in your neighborhood? Case managers? Church? Homeless shelter? Daycare? School?
What was supportive to you about that experience?

3. [If they answered a flyer]

Thinking about the day you saw our flyer, tell me what was going on for you when you decided to call us. ______________________

-What were you thinking about?
-Where were you when you called?
-Why did you want to call?
-Tell me about that first interaction with ARISE

3.1. [If they answered from the iPad]

Tell me the story of first reading the iPad in clinic. ______________________

- What were you thinking when you read it?
- Tell me about the interaction you had with the research assistant.
- What information were you given before you read the iPad?
- What was your first interaction with ARISE?

4. [If they ever talked with someone in the clinic about their relationship]

Sometimes patients (or parents of pediatric patients) also discuss their relationship with someone on the healthcare team in the clinic.

I am interested in hearing about the day you told someone in the clinic about what was going on in your relationship. Would you walk me through that experience?

Additional prompts:

What were you thinking when you walked into the clinic.
Who did you talk to?
What did that person say to you?
What did you say?
What didn't you say? Why?
Tell me about what you discussed about your relationship with that person.
Why did you choose to talk to someone in the clinic?
What was helpful about what that person said to you?
Looking back now, what would have also been helpful?

---

5. [If they have not talked with someone in the clinic about their relationship]

I'd like to hear about a time you went to the clinic with a health concern and didn't tell anyone about that concern.

Additional prompts:

What do you think were the reasons you didn't talk about your health concern with someone in the clinic?
Looking back now, what do you think would have helped you talk about your concern with someone in the clinic that day?

If too abstract then ask: I'd like to hear about the last time you went to the clinic. Would you walk me through that visit?

Additional prompts:

What were you thinking when you walked into the clinic.
Who did you talk to?
What did that person say to you?
What did you say?
What didn't you say? Why?
Tell me about what you discussed with your doctor.
Why did you choose to talk to your doctor about that concern?
What was helpful about that clinic visit?
What was challenging about that clinic visit?
Looking back now, what would have also been helpful?

---

6. [If they ever talked with the IPV Advocate]

In our survey, we asked if you have ever met with or talked to a domestic violence/IPV Advocate when you were at the clinic. Tell me about how it went when you met with (or talked to) the IPV/DV Advocate at the clinic.

Additional prompts:

Let's go through the timeline of that visit. How did you learn about the IPV Advocate?
What happened next?
What was most helpful to you about talking with the Advocate in clinic?
What do you wish was different about that experience talking with the Advocate in clinic?
Why do you think other woman should, or shouldn't, talk to an IPV Advocate in clinic?

7. [If they ever talked with a lawyer] In our survey, we asked if you have ever spoken with a lawyer about problems you are having related to your relationship. You said yes/no. Tell me about your experience meeting with a lawyer. How was this helpful or not helpful to you?

Additional prompts:

Let's go through the timeline of that visit. How did you learn about the lawyer?
What happened next?
What was most helpful to you about talking with the lawyer?

---

8. Relationships and life can be so complicated.

a. Thinking about how complicated your relationship and life has been over the past year. Tell me about a time you think someone in clinic really understood what you were going through in your relationship and your life? What did that person say/do that made you think they understood you?

Additional prompts:

What did that person say/do?
How did you respond?
Walk me through that interaction.
What did you appreciate about the conversation (or thing this person on the healthcare team did for you)?

b. Thinking about how complicated your relationship and life has been over the past year. Tell me about a time you think someone in clinic really did not understand what you were going through in your relationship and life.

Additional prompts:

What did that person say/do?
How did you respond?
Walk me through that experience.
What frustrated or disappointed you about the conversation (or thing this person on the healthcare team did/didn't do for you)?

---

9. Thinking about the past year, tell me about a time you did something to improve your own safety or the safety of your children.

Comment on the safety behaviors checklist. "You said that XXXXX was helpful to you. How was it helpful?"

Additional prompts:

Are there any (other) resources or services that you accessed that made you feel more safe or empowered?
What worked for you to improve your safety or the safety of your children?
What challenges did you have in trying to protect yourself or your children?
Some patients tell us they go swimming, meet a friend for coffee, or play with their dog to feel strong or cope. Are there other things you use to feel strong?

10. Tell me more about a time you did something to cope, protect yourself, heal, or make you feel stronger or more happy?

Additional prompts:

Tell me more about that...
Can you describe more about that to me?
When do you do that? How often? Where?
What do you notice about any changes in your thoughts or feelings when you do that?
